# Natural oil slicks fuel surface water microbial activities in the northern Gulf of Mexico

**DOI:** 10.3389/fmicb.2014.00188

**Published:** 2014-05-08

**Authors:** Kai Ziervogel, Nigel D'souza, Julia Sweet, Beizhan Yan, Uta Passow

**Affiliations:** ^1^Department of Marine Sciences, University of North Carolina Chapel HillChapel Hill, NC, USA; ^2^Lamont-Doherty Earth Observatory, Biology and Paleo Environment, Columbia UniversityPalisades, NY, USA; ^3^Marine Science Institute, University of California Santa BarbaraSanta Barbara, CA, USA; ^4^Lamont-Doherty Earth Observatory, Geochemistry, Columbia UniversityPalisades, NY, USA

**Keywords:** oil slick, Gulf of Mexico, GC600, enzyme activities, carbon cycle, TEP, oil snow

## Abstract

We conducted a series of roller tank incubations with surface seawater from the Green Canyon oil reservoir, northern Gulf of Mexico, amended with either a natural oil slick (GCS-oil) or pristine oil. The goal was to test whether bacterial activities of natural surface water communities facilitate the formation of oil-rich marine snow (oil snow). Although oil snow did not form during any of our experiments, we found specific bacterial metabolic responses to the addition of GCS-oil that profoundly affected carbon cycling within our 4-days incubations. Peptidase and β-glucosidase activities indicative of bacterial enzymatic hydrolysis of peptides and carbohydrates, respectively, were suppressed upon the addition of GCS-oil relative to the non-oil treatment, suggesting that ascending oil and gas initially inhibits bacterial metabolism in surface water. Biodegradation of physically dispersed GCS-oil components, indicated by the degradation of lower molecular weight n-alkanes as well as the rapid transformation of particulate oil-carbon (C: *N* >40) into the DOC pool, led to the production of carbohydrate- and peptide-rich degradation byproducts and bacterial metabolites such as transparent exopolymer particles (TEP). TEP formation was highest at day 4 in the presence of GCS-oil; in contrast, TEP levels in the non-oil treatment already peaked at day 2. Cell-specific enzymatic activities closely followed TEP concentrations in the presence and absence of GCS-oil. These results demonstrate that the formation of oil slicks and activities of oil-degrading bacteria result in a temporal offset of microbial cycling of organic matter, affecting food web interactions and carbon cycling in surface waters over cold seeps.

## Introduction

The northern Gulf of Mexico contains more than 200 hydrocarbon seeps over subseafloor oil reservoirs, releasing up to 1.1 × 10^8^L year^−1^ of oil into the water column. The majority of these seeps are located over the Green Canyon oil reservoir ~200 miles off the Louisiana coast (MacDonald et al., 2002). Ascending hydrocarbon bubbles in that area rapidly dissolve into the bottom water contributing to the isotopically “old” deep water dissolved organic carbon pool (Wang et al., 2001). Depending on bubble size and upwelling currents, oil bubbles can also reach the surface where they form oil microlayers (hereafter referred to as oil slicks) that can grow up to 10 km in length (MacDonald et al., 2002). Evaporation of the volatile components of the surface oil may be rapid (Solomon et al., 2009). Oil slick compounds that are not subject to immediate evaporation likely undergo further weathering processes at the sea surface (MacDonald et al., 2002). Despite the importance of such processes for carbon fluxes and food web interactions, the fate of oil slick residues is not well understood.

Biological oil weathering facilitated by specialized heterotrophic microbial communities plays a key role in the fate of oil-carbon in the ocean (Head et al., 2006). Oil-degrading bacteria often produce large amounts of exopolymeric substances (EPS) to emulsify crude oil (Gutierrez et al., 2013). Enhanced production of bacterial EPS in oil-contaminated surface waters during the Deepwater Horizon (DwH) oil spill in the northern Gulf of Mexico in 2010 led to the formation of mucus webs that in turn accelerated the formation of oil-rich marine snow (Passow et al., 2012). After losing their buoyancy, sinking oil snow resulted in the large sedimentation event of surface oil, now known as the “Dirty Blizzard,” representing a major pathway of DwH surface oil to the seafloor (Hollander et al., 2013; Passow, 2013, 2014). In addition to acting as the glue for oil snow, bacterial EPS may also stimulate activities of heterotrophic bacterial communities not directly involved in primary oil degradation (McGenity et al., 2012). We found evidence for such a bacterial oil degradation cascade in roller tank experiments with oil-contaminated surface water from the DwH site (Ziervogel et al., 2012).

Following the procedure of Ziervogel et al. (2012), we conducted a series of roller tank experiments amending surface seawater with oil slick from the Green Canyon area. These experiments examined whether bacterial transformation of oil slick components triggers the formation of oil snow and subsequent sinking of oil-carbon at the investigated site. Given that ascending oil from the Green Canyon reservoir has been characterized as highly biodegraded (Wang et al., 2001; MacDonald et al., 2002), we conducted supplementary experiments with unaltered pristine oil to acknowledge the role of biological and chemical weathering on the formation of oil snow. We monitored particle formation, dynamics of n-alkanes as well as the particulate and dissolved organic matter pool along with heterotrophic bacterial activities during onboard incubations lasting 4 days. We determined transparent exopolymeric particles (TEP) as a measure for particulate EPS, which often acts as the glue for marine snow (Passow, 2002). To monitor oil snow formation over an extended time period, we also conducted longer term roller tank experiments over 41 days in the home laboratory.

## Materials and methods

### Oil slick sampling

**Figure 1:**
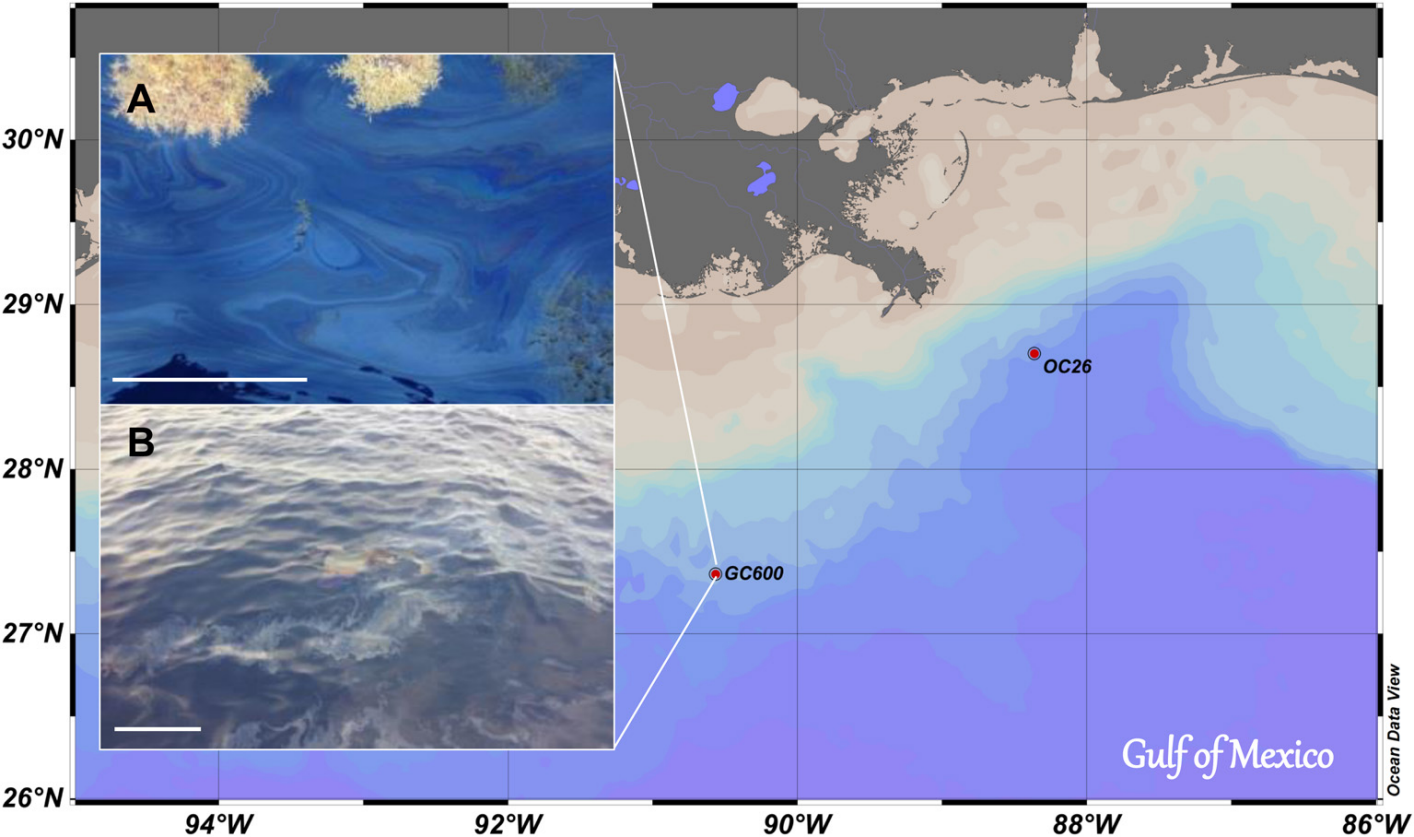
**Map of investigation area**. Oil slick samples were taken at GC 600. Sinking particles for SW+particles and SW+particles+oil treatments were sampled near the bottom of OC 26. **(A)** Oil slick with *Sargassum*; **(B)** Oil slick very similar to the one sampled for this study (Picture taken by V. Asper, USM). Scale bars are 30 cm.

An oil slick-seawater sample was taken at the sea surface over a hydrocarbon seep (GC600; 27° 21.79′N, 90° 34.65′W; water depth: 1200 m; Figure 1) in September 2012 during the RV Endeavor cruise EN515. A clean HDPE container was carefully lowered from the deck of the vessel into the slick to sample ~5 L of an oil slick-seawater mixture that was then filled into a clean cooler (volume: 25 L) on deck. This was repeated four times to sample a total of about 20 L of the oil slick-seawater mixture. Within minutes after sampling the oil formed a microlayer slick at the surface of the cooler water; ~15 L of the excess seawater underlying the oil slick were then released through the drain plug of the cooler. Aliquots of the remaining 5 L of the oil slick-seawater mixture (hereafter referred to as Green Canyon Slick oil, GCS-oil) were added to the roller tanks, and were used for chemical characterization of the GCS-oil (Characterization of GCS-oil).

### Roller tank incubations

#### Green canyon oil experiments (GOE)

A series of short term, onboard roller tank incubations were initiated shortly after sampling of the GSC-oil (Table 1). Acrylic roller tanks (total volume: 1.7 L) were filled with surface seawater that was sampled outside the oil slick at the GC600 site, using Niskin bottles (water depth: 5 m; temperature: 29.5°C; Chlorophyll *a* < 0.2 μg L^−1^). The GOE experiment consisted of four different live treatments, each in triplicate, and one set of killed control tanks containing UV radiated surface seawater. One set of the live treatments contained seawater only, another was supplemented with GCS-oil (1: 43 oil: seawater, v/v). A third and fourth treatment, with and without oil, respectively, additionally contained 10 mL of a particle slurry consisting of planktonic particles collected with an unfixed marine snow trap and *in situ* bottom water. The trap collecting the particle slurry was deployed at 80 m above the seafloor for a period of 6 months at a site ~140 nm to northeast of GC600 (OC26: 28° 44.20′N, 88° 23.23′W; water depth: 1500 m; Figure 1) and recovered 3 days before the start of the roller tank incubation. A qualitative microscopical examination of the particles revealed mainly diatom frustules, fecal pellets and clay minerals. The organic matter content of the particle slurry was low (organic matter to dry weight ratio: 2%). As intended, the addition of the particle slurry promoted coagulation and thus aggregate formation in the tanks (hereafter referred to as macro-aggregates).

**Table 1:** **Experimental set-up of the two onboard roller tank incubations**.

**Experiment ID (date; days of incubation)**	**Treatment ID**	**Treatment description**	**Treatment ratios**	**No of tanks**
Green Canyon oil experiment: GOE (September 2012; 4 days)	SW	Visually uncontaminated surface seawater	unaltered	3
	SW+oil	SW amended with Green canyon oil slick (GCS-oil)	25 mL oil slick: 1075 mL SW	3
	SW+particles	SW amended with particles from marine snow camera trap	10 mL particle slurry: 1090 mL SW	3
	SW+particles+oil	SW amended with particles from marine snow camera trap and GCS-oil	25 mL oil slick: 10 mL particle slurry: 1065 mL SW	3
	SW control	UV radiated SW	Unaltered	3
Pristine oil experiment: POE (June 2012; 3 days)	SW	Visually uncontaminated surface seawater	Unaltered	2
	SW+oil	SW amended with Louisiana crude oil (LA-oil)	1 mL oil: 1099 mL SW	1
	SW control	Autoclaved SW	Unaltered	2
	SW control+oil	Autoclaved SW with LA-oil	1 mL oil: 1099 mL control SW	2

The tanks were filled to the 1.1 L mark; we deliberately left a headspace that led to the formation of an oil slick at the seawater-headspace interface in the oil treatments. The tanks were incubated on a roller table in the dark at a rotation speed of 2.4 rpm and ambient temperature (~20–25°C) for 4 days. Tanks rotation introduced mildly turbulent water mixing, simulating conditions at the sea surface.

One tank per treatment was sampled prior to the start of the incubation (Day 0) as well as after 2 and 4 days, and analyzed for the parameters described below. Tanks were removed from the roller table and placed upright on the bench for sampling. Samples were carefully collected with a 10 mL glass pipette and filled into a clean glass beaker. Special care was taken to avoid transferring oil from the surface slick to the sample water. In the presence of macro-aggregates >1 mm, two separate subsamples were collected: (1) the water overlying all large particles that had settled to the bottom of the tank (surrounding seawater; SSW), and (2) a slurry of macro-aggregates and a known volume of SSW (aggregate slurry; Agg). All analytical parameters are expressed as a concentration or rate per total tank volume (i.e., 1.1 L), accounting for the smaller total volume of the aggregate slurry relative to the surrounding tank water and making direct comparisons and budgeting possible.

An additional roller tank experiment over 41 days was conducted after returning to the home laboratory to study oil snow formation for an extended time that exceeded the period of oil snow formation during the DwH oil spill (~1 month) (Passow et al., 2012). Two roller tanks were filled with either 800 mL of GC600 surface seawater amended with 80 mL of GCS-oil, or 800 mL surface seawater only. The tanks were incubated on a roller table at a rotation speed of 2.4 rpm at low light (<30 μmol m^−2^ s^−1^; 12: 12 h light: dark cycle) and 14°C. Every 1–3 days, the tanks were inspected for macro-aggregates formation without interrupting the rolling motion of the tanks.

#### Pristine oil experiments (POE)

In addition to the GOE experiments with GCS-oil, we also conducted roller tank incubations with surface seawater and pristine oil onboard the RV Endeavor cruise EN510 in June 2012 as well as in the home laboratory (Table 1). The onboard roller tank incubations had surface seawater collected with Niskin bottles at GC600 (27° 21.78′N, 90° 33.88′W; water depth: 5 m; temperature: 28.7°C; Chlorophyll *a* < 0.2 μg L^−1^) either amended with Louisiana crude oil (LA-oil; WP681 from Fisher Scientific) at a ratio of 1: 1100 (v/v) or unamended. Autoclaved surface seawater with and without LA-oil served as killed controls. Incubation time of the onboard Pristine Oil Experiment (POE) was 3 days, and the tanks were incubated under the same conditions and analyzed at day 0 and day 3 for the same parameters as in GOE. Data for day 0 from the oil amended seawater tank is missing.

As for GOE, we conducted a 41-days POE experiment in the home laboratory with two roller tanks filled with 1000 mL of GC600 surface seawater amended with 1 mL of Macondo crude oil (provided by J Short, JWS Consulting LLC, LSU ID 2010158-12, MC-252 Source oil 5/20/10). The tanks were incubated on a roller table under the same conditions as described for the longer term GOE. Every 1–3 days, the tanks were inspected for macro-aggregate formation without interrupting the rolling motion of the tanks.

### Analytical methods

#### Characterization of GCS-oil

***Hydrocarbon extraction and fractionation***. Oil slick water from the cooler (GCS-oil) as well as roller tank water from the two GCS-oil treatments at day 4 (SW+oil; SW+particles+oil) were filled into separate 2.5-L amber glass bottles, fixed with Sodium Azide (0.02% final conc.), and stored in the dark at 4°C until extraction. Hydrocarbon extraction was conducted via passage through an Empore C18 solid phase extraction disk installed in a vacuum apparatus after the samples were acidified to a pH of ~2.7 for optimal hydrocarbon adsorption to the pre-conditioned disk. Hydrocarbons were eluted from the disks with a mixture of hexane and acetone at a ratio of 7: 3 (v/v), then passed through an anhydrous Na_2_SO_4_ column and solvent-exchanged to hexane under a steady stream of nitrogen. Samples were centrifuged for 10 min at 10,000 rpm in 2 mL Teflon centrifuge tubes, and the hexane-insoluble fraction was discarded. To purify and separate the aliphatic and aromatic fractions, we performed a fractionation with two silica gel columns; one column was topped with anhydrous Na_2_SO_4_ for the removal of residual water, the other had copper powder for the removal of elemental sulfur. Two elutions were performed on each column, with 15 mL of hexane followed by 15 mL of a 1: 1 mixture of hexane and dichloromethane (v/v). Following column fractionation, the extracts were concentrated under nitrogen and stored at 4°C until further analysis.

***GC × GC analysis***. Fractionated hydrocarbon extracts were analyzed using a Leco comprehensive two-dimensional gas chromatograph—time-of-flight-mass spectrometer (GC × GC-TOF). The samples were injected in splitless mode with the inlet temperature at 300°C. The first-dimension column oven in the GC × GC was held at 60°C for 1 min, then gradually increased at a rate of 1.5–315°C, where it was held for 15 min. The thermal modulator temperature offset was set to 55°C above the first-dimension column (Restek Rxi-1MS column, 20 m length, 0.18 mm I.D., 0.18 μm film thickness), and the temperature offset of the secondary column (50% phenyl polysilphenylene-siloxane column, SGE BPX50, 1 m length, 0.1 mm I.D., 0.1 μm film thickness) was set to 30°C above the first-dimension column oven. Helium was used as the carrier gas at a constant flow rate of 1.5 mL min^−1^.The thermal modulator hot jet pulse time was 0.6 s with a 6.9 s cool time between stages. Dried air was used to supply the thermal modulator hot and cold jet gas. The detector signal was sampled at 200 spectra second^−1^. The transfer line temperature was 310°C, the source temperature was 200°C, and the acquisition voltage was 1677 V. Analytes were quantified for n-alkanes using commercially available standards from Restek Corp. (Bellefonte, PA) and Sigma-Aldrich (St. Louis, MO).

***Oil fluorescence***. During the onboard GOE, we also monitored relative changes in oil fluorescence. Two milliliter of tank water were filled into disposable methacrylate cuvettes and the raw fluorescence signal was measured using the crude oil module (#7200-063; excitation: 365, emission: 410–600) of the Trilogy Laboratory Fluorometer (Turner). The GCS-oil fluorescence signal, expressed as relative fluorescence units (RFU), was corrected with the signal in the unamended seawater treatment that was always considerably lower compared to the oil containing treatments.

#### Transparent exopolymer particles (TEP)

TEP representing a particulate form of EPS were measured colorimetrically in triplicate samples per tank by filtration of 50–100 mL of tank water onto 0.4 μm polycarbonate filters and subsequent staining with Alcian Blue (Passow and Alldredge, 1995). The dye solution, which complexes carboxyl and half-ester sulfate reactive groups of acidic polysaccharides was calibrated using Gum Xanthan.

#### Dissolved organic carbon (DOC)

DOC was analyzed in pre-filtered (0.2-μm surfactant free cellulose acetate syringe filters) and acidified (50% phosphoric acid v/v) samples by high temperature catalytic oxidation using a Shimadzu TOC-5000. DOC was not measured in aggregate slurries. Duplicate samples per tank were injected; instrument settings yielded at least three repeated measurements of each sample.

#### Particulate organic carbon and nitrogen (POC, PON)

For POC and PON analysis, duplicate tank water samples of 50–250 mL were vacuum filtered onto pre-combusted GF/F filters. The filters were acidified with 12 M HCl for 12 h to remove inorganic carbon prior to flash combustion to CO_2_ and N_2_ on a Carlo-Erba 1500 Elemental Analyzer, using acetanilide as a standard.

#### Bacterial abundance

Ten milliliter of tank water was fixed with formalin immediately after sampling (2% final conc.) and stored for 8 months in the dark at 4°C before the preparation of microscope slides. Prior to slide preparation, samples were pretreated to break up bacterial aggregates and detach bacteria from particles. We used Tween-80 (0.5% final conc.), EDTA (50 mM final conc.), and 10× PBS pH 7.4 (5.26% final conc.), followed by vortex mixing for 10 min. Samples were then sonicated for 3 min in a water bath (adapted from Suter, 2011, and Crump et al., 1998), and stained according to Lunau et al. (2005) as described below. Samples with no macro-aggregates were also subjected to this treatment, and cells numbers counted in selected samples before and after the treatment revealed an increase of 38–152%. Samples used for enumeration of bacterial micro-aggregates were not subject to any physical or chemical treatments prior to staining.

A known volume of each pretreated sample was drawn through a 25 mm diameter 0.2 μm pore, black polycarbonate filter (Millipore, type GTPB) using low vacuum. The filters were transferred to clean microscope slides. Ten microliter of a freshly prepared mounting medium containing 50% glycerol in 1× PBS at pH 7.4, ascorbic acid (1% final conc. v/v), and SYBR green I stain (0.45% final conc. v/v) was placed in the middle of a cover slip (25 mm × 25 mm) and inverted onto the filter. The slide was then placed in the dark at 4°C, until the weight of the cover slip dispensed the stain evenly across the filter. Bacterial cells and micro-aggregates were counted with a Nikon Labophot-2 epifluorscence microscope with blue light excitation at 1000× and 200× magnification respectively. For bacterial cell counts, a minimum of 200 cells were enumerated within a grid of fixed dimensions across each filter. Micro-aggregates were categorized into three distinct size classes (5–40, 40–80, >80 μm) using an ocular grid. Unless the abundance of micro-aggregates was low, a minimum of 100 aggregates were enumerated within a grid of fixed dimensions across each filter. Bacteria cell counts in tank water samples from the onboard POE were counted by flow cytometry (Accuri C6) using SYBR-GREEN I as a stain.

#### Bacterial enzymatic activities

Potential hydrolytic activities of carbohydrate- and peptide-hydrolyzing enzymes were measured using 4-MUF-β-D-glucopyranoside and L-leucine-MCA hydrochloride, respectively, as fluorogenic substrate analogs according to Hoppe (1983). Three mL of tank water were added to triplicate disposable methacrylate cuvettes containing fluorogenic substrate analogs at saturation levels (300 μM for L-leucine-MCA hydrochloride; 333 μM for 4-MUF-β-D-glucopyranoside). Cuvettes were incubated under the same conditions as the roller tanks. Fluorescence was measured immediately after sample addition and at two additional time points over the course of 24 h under buffered conditions (20 nM borate buffer; pH 9.2) using a Turner Biosystems TBS-380 fluorometer (excitation/emission channels set to “UV”; 365 nm excitation, 440–470 nm emission). Fluorescence changes were calibrated using standard solutions of 4-methylumbelliferone and 4-methylcoumarin in tank water, and used to calculate hydrolysis rates expressed as cell-specific rates. All of the killed control tanks in GOE and POE showed only minor changes in fluorescence over time possibly due to abiotic substrate hydrolysis. Fluorescence changes in the killed control treatments were used to correct enzymatic hydrolysis rates in the respective live treatments.

### Statistical analysis

Results of analytical parameters are given as average values ± standard deviation. Differences between two average values were analyzed using the Student's *t*-test; differences between three or more average values were assessed using an analysis of variance (One-Way ANOVA) with Tukey HSD *post-hoc* pairwise comparisons of means at the 5% significant level (*p* = 0.05). All statistical analysis was performed in Excel® using the data analysis tool pack.

## Results

### GCS-oil dynamics during roller tank incubations

**Figure 2:**
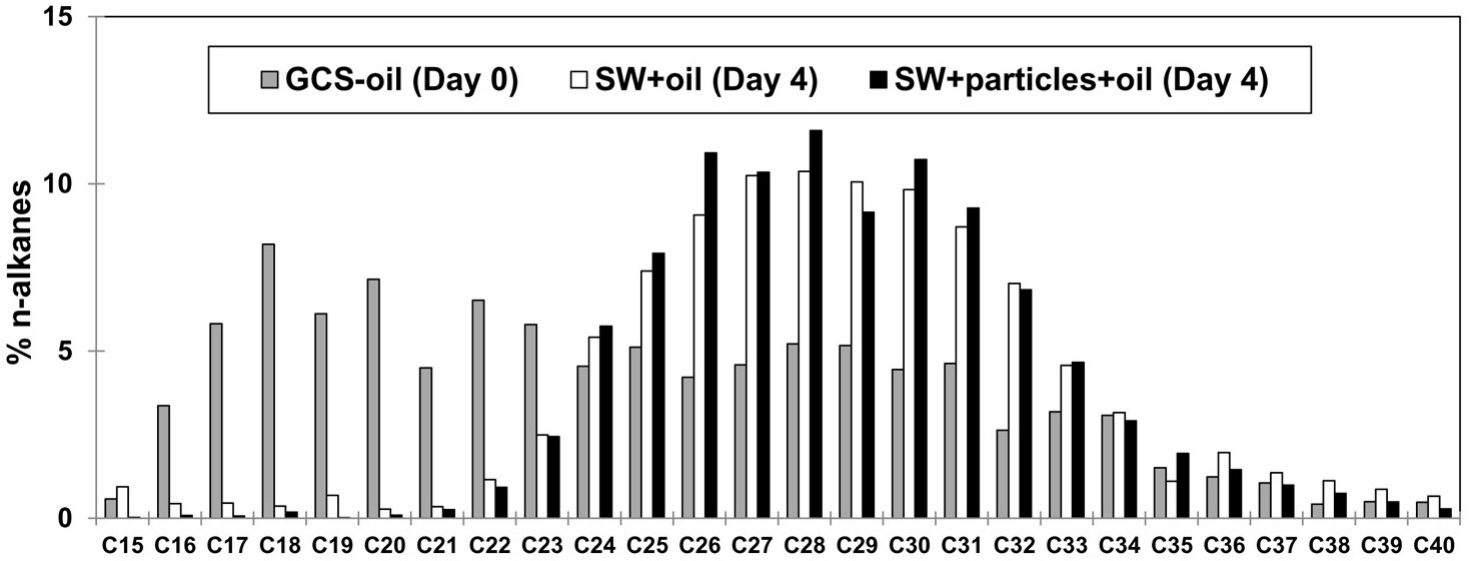
**Chemical characterization of GCS-oil**. Relative distribution of n-alkanes before (Day 0) and after the roller tank incubations (Day 4). Note that all three samples were mostly depleted of <C_15_ alkanes, probably due to dissolution during ascending of the oil from the seep.

The C_16_–C_34_ components in the initial GCS-oil sample ranged between 3 and 8% of the total n-alkane pool (Figure 2). At the end of both GCS-oil incubations (SW+oil and SW+particles+oil), levels of C_16_–C_21_ components decreased by up to one order magnitude compared to the initial sample. All three samples were mostly depleted of <C_15_ alkanes, probably due to dissolution during ascending of the oil from the seep.

The initial fluorescence signals in both GCS-oil treatments were on average at 23,072 ± 2538 RFU, decreasing to 50% at day 2 in both treatments. Only 27% and 7% of the initial fluorescence signal were detected at day 4 resulting in a linear decrease in oil fluorescence over time in SW+oil (*r*^2^ = 0.99) and SW+particles+oil (*r*^2^ = 0.97), respectively.

### Formation of macro-aggregates

Macro-aggregate formation was only observed in the presence of the particle slurry during the onboard GOE. By day 4 a single aggregate per tank (diameter: ~2 mm) had formed. The presence or absence of GCS-oil had no a visible impact on the formation of macro-aggregates. No particles >1 mm formed in any of the treatments of the longer term GOE, but particles <1 mm appeared in the presence of GCS-oil at day 19. These particles remained unchanged until the end of the 41 days incubation. No particles of any size were visible in any of the POE experiments.

### Transparent exopolymer particles (TEP)

TEP concentrations in the onboard GOE seawater treatment (SW) doubled between days 0 and 2, followed by a decrease to initial levels at day 4 [*F*_(2, 6)_ = 6.87, *p* < 0.05; Table 2]. In contrast TEP remained low until day 2 in the presence of GCS-oil (SW+oil), and subsequently increased by a factor of 4 until the end of the incubation [F_(2, 6)_ = 11.5, *p* < 0.01]. The addition of the particle slurry (SW+particles) led to almost 5 times higher initial TEP concentrations compared to initial SW levels (Student's *t*-test, *p* < 0.05). The amount of TEP in macro-aggregates (Agg), in the presence or absence of GCS-oil, was similar to that in the SSW; at day 4 in the SW+particles treatment, the amount of TEP incorporated in the Agg fraction was lower than in SSW [F_(4, 10)_ = 6.7, *p* < 0.01]. TEP in POE more than doubled during the time course of the non-oil (SW) incubation with similar levels in SW and SW+oil at day 3 [*F*_(2, 5)_ = 38.2, *p* < 0.001].

**Table 2:** **Dynamics of the organic carbon pool**.

**Experiment**		**GOE**					**POE**	
**Day**		**0**	**2**		**4**		**0**	**3**
**Fraction**		**SSW**	**SSW**	**Agg**	**SSW**	**Agg**	**SSW**	**SSW**
TEP	SW	328 ± 397; *B*	1181 ± 170; *A*	–	522 ± 274; *B*	–	478 ± 42; *B*	1137 ± 116; *A*
	SW+oil	180 ± 42; *B*	287 ± 151; *B*	–	1043 ± 385; *A*	–	n.d.	1444 ± 151; *A*
	SW+particles	1541 ± 21; *A*	1006 ± 112; *B*	982 ± 79; *B*	980 ± 150; *B*	417 ± 55; *C*	No exp.	No exp.
	SW+particles+oil	944 ± 58; *A*	397 ± 23; *B*	679 ± 114; *AB*	387 ± 154; *B*	523 ± 275; *B*	No exp.	No exp.
DOC	SW	1507 ± 7; *C*	4518 ± 36; *A*	–	1896 ± 109; *B*	–	3548 ± 716; *A*	1776 ± 19; *B*
	SW+oil	1148 ± 66; *B*	2039 ± 59; *A*	–	2630 ± 524; *A*	–	n.d.	1315± 59; *B*
	SW+particles	2203 ± 628;*B*	1818 ± 100; *AB*	–	3006 ± 109; *A*	–	No exp.	No exp.
	SW+particles+oil	1433 ± 30; *B*	1906 ± 130; *AB*	–	2816 ± 29; *A*	–	No exp.	No exp.
POC	SW	117 ± 6; *B*	247 ± 6; *A*	–	241 ± 11; *A*	–	121^#^	193 ± 18
	SW+oil	867 ± 15; *A*	595 ± 116; *AB*	–	356 ± 58; *B*	–	n.d.	312 ± 25
	SW+particles	310 ± 3; *AB*	172 ± 2; *D*	383 ± 53; *A*	227 ± 0.1; *CD*	297 ± 8; *BC*	No exp.	No exp.
	SW+particles+oil	1032 ± 83; *A*	305 ± 111; *B*	401 ± 31; *B*	249 ± 7; *B*	287 ± 32; *B*	No exp.	No exp.
C/N	SW	8.3 ± 0.6; *n.s*.	8.9 ± 0.9; *n.s*.	–	6.2 ± 0.4; *n.s*.	–	10.5^#^	7.2± 0.4
	SW+oil	40.9 ± 2.5; *A*	16.2 ± 1.3; *B*	–	12.1 ± 1.7; *B*	–	n.d.	10.6 ± 0.4
	SW+particles	7.7 ± 0.3; *BC*	6.6 ± 0.1; *C*	9.5 ± 0.1; *B*	9 ± 0.8; *B*	14.3 ± 0.8; *A*	No exp.	No exp.
	SW+particles+oil	34.2 ± 3.3; *A*	13.7 ± 2.8; *B*	14.1 ± 0.4; *B*	13.2 ± 0.6; *B*	12.2 ± 0^*^; *B*	No exp.	No exp.

*TEP (μg GXeq tank^−1^), DOC (μg tank^−1^), POC (μg tank^−1^)*.

*SSW, surrounding seawater; Agg, macro-aggregates*.

*Letters indicate the results of the post-hoc analysis following ANOVA (p < 0.05); Concentrations with the same letters are statistically indistinguishable from one another*.

*n.d. means not determined, no exp. means no experiment*.

# single measurement;

* *number <0.1*.

### Dissolved and particulate organic carbon (DOC, POC)

DOC concentration in the onboard GOE seawater treatment (SW) increased between days 0 and 2 by a factor of 3, followed by a decrease to initial levels at the end of the incubation [*F*_(2, 7)_ = 1519.9, *p* < 0.001; Table 2]. DOC in the presence of GCS-oil doubled until day 2, remaining at this high level until day 4 [*F*_(2, 9)_ = 23.6, *p* < 0.001]. DOC in both particle slurry treatments gradually increased until day 4 [SW+particles: *F*_(2, 11)_ = 9.1, *p* < 0.005; SW+particles+oil: *F*_(2, 8)_ = 5.9, *p* < 0.001]. In POE, DOC decreased by a factor of 2 in SW until day 3, reaching similar levels in the presence and absence of LA-oil at day 3 [*F*_(2, 19)_ = 70.7, *p* < 0.001].

Levels of POC in the GOE seawater tank (SW) more than doubled between days 0 and 2, remaining constant until the end of the incubation [*F*_(2, 3)_ = 117.1, *p* < 0.001; Table 2]. The addition of GCS-oil led to a 7-fold higher initial POC concentration in SW+oil compared to SW at day 0 (Student's *t*-test, *p* < 0.05). POC in SW+oil gradually decreased until day 4 [*F*_(2, 3)_ = 13.2, *p* < 0.05]. The particle slurry treatment in the absence of GCS-oil (SW+particles) also had a higher initial POC concentration compared to SW (Student's *t*-test, *p* < 0.05). At day 2, POC concentration in macro-aggregates (Agg) was a factor of 2 higher than in SSW; subsequently POC in SSW slightly increased leading to similar levels in Agg and SSW at the end of the incubation [*F*_(4, 5)_ = 23.1, *p* < 0.005]. Highest initial POC levels of all four treatments were measured in SW+particles+oil [*F*_(3, 4)_ = 215.1, *p* < 0.001]. Following macro-aggregate formation, POC in Agg and SSW remained at the same levels at days 2 and 4 [F_(4, 5)_ = 50.5, *p* < 0.001]. POC concentrations in POE increased by factors of 1.5 in SW until day 3; SW+oil at day 3 had 2.5 higher levels of POC compared to the initial SW level (no statistical analysis due to lack of replicates at day 0).

During the GOE experiment, ratios of C: N in SW were statistically indistinguishable from one another, ranging between 6.2 and 8.9 (Table 2). The addition of GCS-oil led to an increase of the C: N ratios by a factor of 4 relative to the non-oil treatments. C: N ratio in SW+oil gradually dropped from 41 at day 0 to 12 at day 4 [F_(2, 3)_ = 162.5, *p* < 0.001]. SW+particels+oil had initial C: N ratios of 34, decreasing until day 2 by a factor of 2.5, and remaining at the same levels in SSW and Agg until the end of the incubation [*F*_(4, 5)_ = 46.8, *p* < 0.001]. In SW+particles, C: N ratios were low throughout the first 2 days of the incubation, reaching highest ratios in Agg at day 4 [*F*_(4, 5)_ = 33.9, *p* < 0.001]. In POE, ratios of C: N were 10.5 and 7.2 in SW at day 0 and day 3, respectively, and 10.6 in SW+oil at day 3.

### Bacterial cell numbers and micro-aggregate formation

Initial bacterial cell numbers in all four GOE treatments were in the same order of magnitude, ranging between 2.5 * 10^8^ tank^−1^(SW+particles+oil) and 5.6 * 10^8^ tank^−1^(SW+particles). On day 2, cell numbers in SW and SW+oil were up to 3.5 times higher compared to day 0, thereafter remaining at that higher level in SW (Table 3). In contrast, bacterial numbers in SW+oil decreased to initial levels until day 4. Fewer cells were associated with macro-aggregates compared to SSW in the absence of GCS-oil (SW+particles). Bacterial numbers associated with Agg in SW+particles+oil treatments were similar (day 2) or higher (day 4) compared to those in the SSW. Bacteria cells in both POE treatments increased by up to one order of magnitude during the 3 days incubation.

**Table 3:** **Bacterial abundance and cell-specific enzymatic activities**.

**Experiment**		**GOE**					**POE**	
**Day**		**0**	**2**		**4**		**0**	**3**
**Fraction**		**SSW**	**SSW**	**Agg**	**SSW**	**Agg**	**SSW**	**SSW**
Bact. cells	SW	4.0	14.1	–	11.7	–	6.4	10.6
	SW+oil	3.5	9.9	–	2.5	–	n.d.	16.0
	SW+particles	5.6	3.4	1.5	7.4	1.1	No exp.	No exp.
	SW+particles+oil	2.5	1.8	1.4	1.2	3.9	No exp.	No exp.
β-glu	SW	52 ± 2; *C*	136 ± 10; *A*	–	74 ± 25; *B*	–	6 ± 0^*^; *B*	8 ± 1; *B*
	SW+oil	11 ± 1; *C*	42 ± 2; *B*	–	138 ± 6; *A*	–	n.d.	53 ± 14; *A*
	SW+particles	55 ± 1; *D*	165 ± 21; *B*	212 ± 0; *A*	45 ± 11; *D*	102 ± 6; *C*	No exp.	No exp.
	SW+particles+oil	27 ± 2; *C*	113 ± 5; *B*	257 ± 7; *A*	96 ± 26; *B*	119 ± 17; *B*	No exp.	No exp.
pep	SW	177 ± 5; *B*	788 ± 209; *A*	–	995 ± 34; *A*	–	26 ± 0.2; *C*	55 ± 1; *B*
	SW+oil	102 ± 2; *B*	150 ± 15; *B*	–	1749 ± 73; *A*	–	n.d.	164 ± 4; *A*
	SW+particles	147 ± 12; *E*	3051 ± 62; *C*	10093 ± 125; *B*	1374 ± 19; *D*	12872 ± 401; *A*	No exp.	No exp.
	SW+particles+oil	173 ± 10; *D*	1149 ± 35; *C*	2112 ± 50; *B*	2465 ± 10; *A*	1140 ± 17; *C*	No exp.	No exp.

*Bacterial cells (× 10^−8^ tank^−1^), β-glucosidase (β-glu, amol cell^−1^ hr^−1^), peptidase (pep, amol cell^−1^ hr^−1^)*.

*n.d. means not determined, no exp. means no experiment*.

* *number <0.1*.

**Figure 3:**
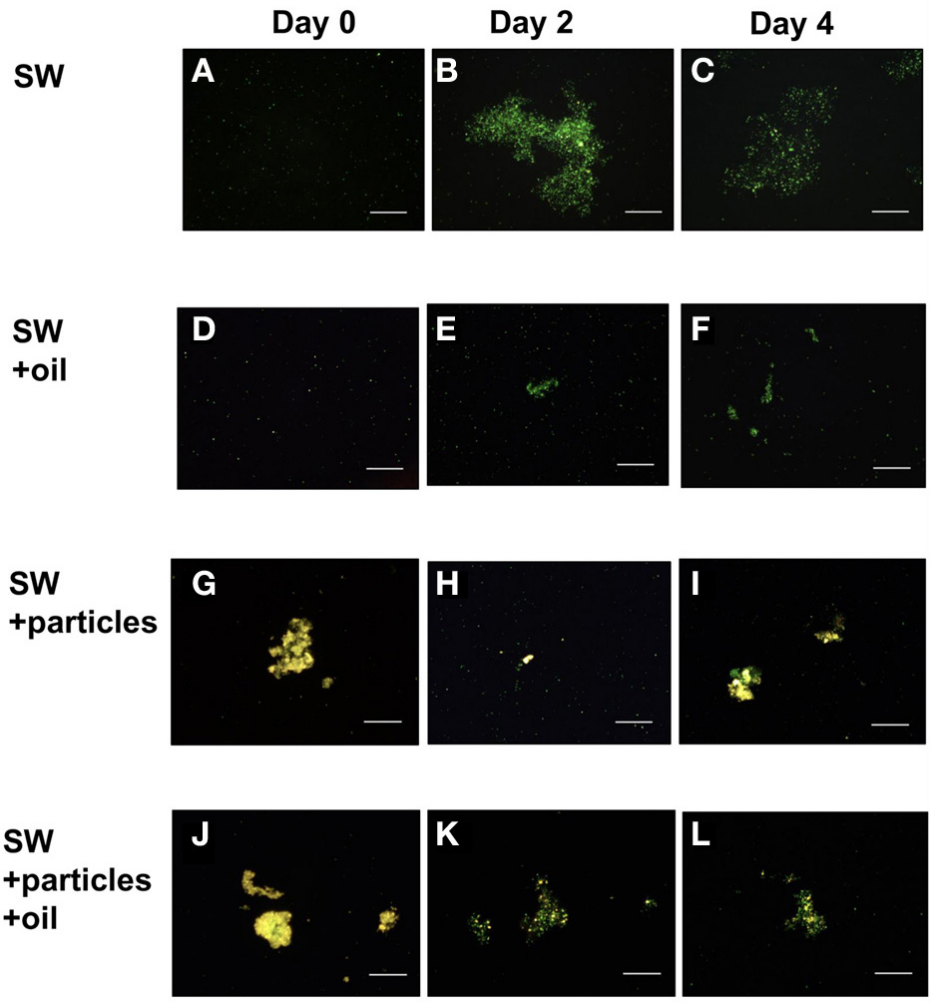
**Bacterial micro-aggregates in GOE**. Epilfluorescence microscopy of untreated tank water samples at days 0 **(A,D,G,J)**, 2 **(B,E,H,K)**, and 4 **(C,F,I,L)** reveal the development of bacterial micro-aggregates in the absence of oil at days 2 **(B)** and 4 **(C)**. Tank water samples amended with GCS-oil lack micro-aggregates. All micrographs were imaged at 200× magnification, and scale bars are 50 μm.

**Figure 4:**
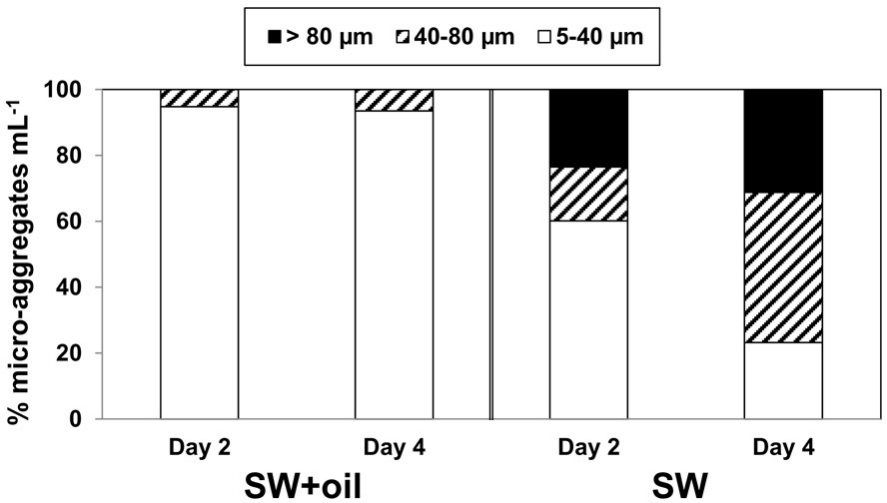
**Size distributions of bacterial micro-aggregates in GOE**. Percentage distribution of three aggregate size classes in the seawater without GCS-oil (SW), and with GCS-oil (SW+GCS-oil).

Epifluorescence microscopy of samples not subject to physical or chemical dis-aggregation treatments revealed the formation of bacterial micro-aggregates in SW and SW+oil at day 2 (Figure 3). Bacterial micro-aggregates in SW increased in size between days 2 and 4 (Figure 4); in the oil amended tank (SW+oil), micro-aggregates remained smaller at days 2 and 4 compared to SW. In seawater samples amended with particles, we observed yellow fluorescing material that in the presence of GCS-oil appeared to be heavily colonized by bacteria on days 2 and 4 relative to SW+particles (Figures 3K,L).

### Bacterial enzymatic activities

Cell-specific glucosidase activities in the GOE seawater treatment (SW) peaked at day 2 and were lowest at day 0 (Table 3). The addition of GCS-oil lowered the initial cell-specific glucosidase activities in SW+oil by a factor of 5 relative to initial SW levels (Student's *t*-test, *p* < 0.001). Cell-specific glucosidase activity in SW+oil subsequently increased until day 4 [*F*_(2, 6)_ = 842.7, *p* < 0.001]. Macro-aggregate associated activities in both particles treatments (SW+particels, SW+particles+oil) were generally higher compared to the SSW [SW+particels: *F*_(4, 9)_ = 112.2, *p* < 0.001; SW+particles+oil: *F*_(4, 9)_ = 78.3, *p* < 0.001]. Cell-specific glucosidase activities in POE remained constant in the seawater treatment; one order of magnitude higher activities were found in SW+oil compared to the initial SW level [*F*_(2, 3)_ = 11.3, *p* < 0.05].

Cell-specific peptidase activity increased within the first 2 days of the GOE seawater treatment (SW) by a factor of 4.5, remaining at that level until day 4 (Table 3). The addition of GCS-oil lowered initial cell-specific peptidase activities by a factor of up to 2 in SW+oil compared to SW (Student's *t*-test, *p* < 0.001). Cell-specific peptidase activities in SW+oil remained at the same level between days 0 and 2, and increased by one order of magnitude until the end of the incubation [*F*_(2, 3)_ = 868.4, *p* < 0.001]. The formation of macro-aggregates in the absence of GCS-oil (SW+particles) led to cell-specific peptidase activities in Agg at days 2 and 4 that were one to two orders of magnitude higher compared to SSW [*F*_(4, 10)_ = 26.74, *p* < 0.001]. In contrast cell-specific peptidase activity in Agg in the presence of macro-aggregates and GCS-oil (SW+particles+oil) were one order of magnitude lower compared to Agg in the non-oil treatment. Highest cell-specific peptidase activities in SW+particles+oil were found in SSW at day 4 [*F*_(4, 10)_ = 2904.7, *p* < 0.001]. In POE, cell-specific peptidase activity in SW doubled between days 0 and 2, and peptidase activities in the presence of oil were about 3 times higher compared to SW at day 3 [*F*_(2, 3)_ = 868.4, *p* < 0.001].

## Discussion

Our experiments were designed to test whether oil slick residues from natural oil and gas seeps over the Green Canyon oil reservoir (Figure 1) form oil-rich marine snow. Such oil snow would provide a transport pathway to the sea floor for oil-carbon, similar to the oil snow that formed during the early stages of the DwH oil spill in 2010 (Passow et al., 2012; Ziervogel et al., 2012). The observed macro-aggregates in SW+particles and SW+particles+oil formed due to coagulation of the added particles, and thus differed appreciably from the DwH oil snow that formed as a result of enhanced bacterial mucus production and in the absence of suspended particles (Passow, 2014). Despite the lack of oil snow formation, the addition of GCS-oil to surface water triggered a specific bacterial metabolic response that affected the timescales of bacterial cycling of carbon in our roller tank incubations.

### Effects of GCS-oil on carbon cycling in the absence of macro-aggregates

GCS-oil formed a microlayer at the tank water-headspace interface similar in thickness to the one observed in the field (Figure 1). The initial oil fluorescence as well as the C: N ratios of the POM pool in the GCS-oil tanks compared to the non-oil treatments (Table 2) indicate that some fraction of the GCS-oil instantly dispersed into the water underneath the surface layer. Turbulent conditions during filling of the GCS-tanks possibly dispersed the oil into μm-sized droplets (e.g. Li and Garrett, 1998). Physically dispersed oil did not affect the DOC pool (Table 2), indicating that most of the water soluble fraction of the GCS-oil was removed during its ascent from the seep at 1200 m. This agrees with field observations at Green Canyon seeps, revealing a rapid dissolution of the water soluble fraction of the seep oil into bottom waters (Wang et al., 2001).

Dispersed oil in the tank water was more available to biodegradation compared to the oil slick that persisted until the end of the incubation. The rapid decrease of C: N ratios, POC concentrations, and oil fluorescence accompanied with an increase in DOC in SW+oil (Table 2) point to the transformation of oil-carbon into the DOC pool. This transformation was facilitated by heterotrophic bacteria capable of degrading the lower molecular weight n-alkanes of the dispersed oil throughout the GCS-oil incubations (Figure 2). Similar biodegradation patterns of n-alkanes were observed during the early stages of the DwH oil spill in the deep-water hydrocarbon plume where C_13_–C_26_ n-alkanes had half-lives of 1–6 days (Hazen et al., 2010).

**Figure 5:**
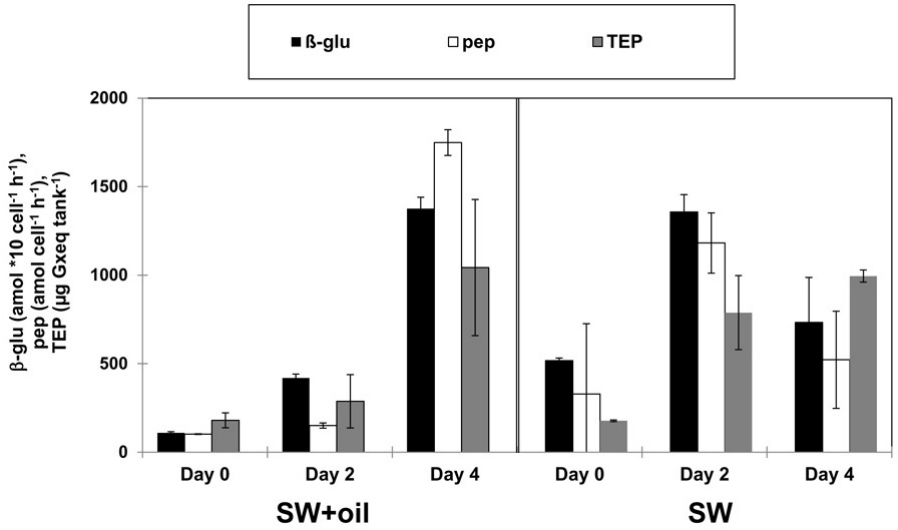
**TEP and enzymatic activities in GOE**. Enzymatic activities of β-glucosidase (β-glu) and peptidase (pep) show the same temporal patterns than TEP (transparent exopolymeric particles) in the tank with GCS-oil (SW+GCS-oil) and in the unamended tank (SW). Error bars indicate standard deviations of *n* = 3.

Biodegradation of oil in the ocean often leads to the production of peptide- and carbohydrate-rich organic macromolecules as degradation byproducts (Hazen et al., 2010) and bacterial EPS (Gutierrez et al., 2013). Such metabolic byproducts from primary oil degradation can stimulate activities of non-oil degrading heterotrophic bacterial communities (McGenity et al., 2012). GeoChip analysis of DNA in the deep-water hydrocarbon plume that formed in the early stages of the DwH oil spill revealed high levels of genes associated with the degradation of high molecular weight carbohydrates compared to non-plume associated deep waters (Lu et al., 2012). In addition leucine-aminopeptidase and β-glucosidase activities were elevated in deep waters inside compared to outside the hydrocarbon plume (Ziervogel and Arnosti, in press). Similar to the bacterial dynamics during the DwH oil spill, the here observed patterns of enzyme activities and TEP (used as indicators for bacterial EPS) also point to a bacterial metabolic cascade of primary oil degraders and secondary consumers. Levels of TEP were in the same range in the GCS-oil and the non-oil treatment; however the different temporal development of TEP formation (Table 2) points to different bacterial metabolic dynamics in the two treatments. Rapid bacterial growth and metabolism after the start of the non-oil incubation (SW) led to enhanced levels of TEP at day 2. TEP in SW served as the organic network resulting in the formation of bacterial micro-aggregates (Figures 3B,C). In contrast, the addition of GCS-oil initially suppressed bacterial metabolism as indicated by the lower enzyme activities at day 0 in the GCS-oil relative to the non-oil treatments (Table 2). This partial inhibition of bacterial metabolism in the SW+oil treatment led to lower TEP production in the first 2 days. However, EPS produced by primary oil degraders accumulated throughout the experiment in the GCS-oil treatments, resulting in peak levels of TEP at day 4. The close correlation between TEP and cell-specific enzymatic activities in both treatments (Figure 5) underlines the role of TEP as an important carbon source for heterotrophic bacteria (Ortega-Retuerta et al., 2010). It also demonstrates the delayed metabolic response of the fraction of the bacterial community not involved in primary oil degradation.

### Effects of GCS-oil on carbon cycling in tank water with macro-aggregates

In addition to TEP formation from bacterial production, TEP was introduced with the particle slurry used for the “+particles” treatments as indicated by initially higher TEP concentrations in SW+particles and SW+particles+oil compared to the non-particle treatments (Table 2). Thus, sticky particles rapidly formed macro-aggregates that were colonized by surface water bacterial communities; it is also possible that bacterial cells attached to the source particles from the marine snow trap were carried over. Bacteria associated with macro-aggregates expressed generally higher enzymatic activities compared to those in SSW (Table 2). Marine aggregates, in general, are hotspots for heterotrophic bacterial activities (Azam, 1998) often expressing higher peptidase compared to β-glucosidase activitiy (Smith et al., 1992). Cell-specific peptidase activity was particularly enhanced in macro-aggregates in the absence of GCS-oil (SW+particles), suggesting that the presence of oil may have suppressed macro-aggregate associated peptidase activities in SW+particles+oil.

Ratios of C: N and POC levels in the Agg and SSW of the GCS-oil treatment indicate that macro-aggregates did not incorporate significant amounts of dispersed oil (Table 2). Nevertheless, some fraction of the GCS-oil possibly absorbed onto mineral particles in the Agg as well as the SSW fraction of the SW+particles+oil treatment. Given that mineral particles were present in the source slurry, we hypothesize that μm-sized mineral-oil complexes formed in the tanks with GCS-oil, acting as hotspots for bacterial oil-degraders in SSW. This would explain why the μm-sized particles in SW+particles+oil appeared to be more colonized by bacteria relative to the ones in the non-oil treatment (Figures 3G–L).

The very similar dynamics of the n-alkane pool in the presence (SW+particles+oil) and absence of particles (SW+oil) (Figure 2) indicate that the formation of macro-aggregates had no measurable effect on the bioavailability of the dispersed oil within the timecourse of our 4-days incubations. Considering fluxes of the C_16_–C_21_ n-alkanes, [i.e., the biodegraded fraction of the dispersed oil (Figure 2)], we found that 7% of this pool was left in SW+oil at day 4 while only 2% of the C_16_–C_21_ n-alkanes remained in SW+particles+oil at the end of the incubations. This suggests higher rates of oil biodegradation in the presence of macro-aggregates (and mineral-oil complexes in the SSW) at similar total bacterial abundances in the treatments with and without particles. Higher oil biodegradation rates could also explain the greater loss of oil-carbon in SW+particles+oil compared to SW+oil: the addition of GCS-oil led to an increase of 700 μg POC per tank in both treatments (i.e., initial POC in oil treatments subtracted by the respective non-oil treatments). At day 4, 15% of the added oil-carbon was left in SW+oil while only 3% of the oil-carbon was left at the end of the treatment with macro-aggregates.

### Conclusions

The lack of oil snow formation in surface seawater over the Green Canyon oil reservoir demonstrates that the fate of oil slick residues stemming from natural deep water seeps in the northern Gulf of Mexico is not driven by sinking oil snow. Fractions of the oil slick, however, may be available for rapid biodegradation, initiating a bacterial metabolic cascade that utilized ~85% of the oil-carbon within our 4-days incubations. This contrasts with previous studies from the Green Canyon area, suggesting that oil slicks contain highly degraded oil components that had only minor effects on biogeochemical fluxes in the surface ocean (Wang et al., 2001; MacDonald et al., 2002). Moreover higher rates of oil biodegradation can be expected in the presence of macro-aggregates formed by coagulation of suspended particles. Concentrations of suspended particles in northern Gulf of Mexico surface waters increase with decreasing distance to the coast. Thus, in addition to UV radiation (MacDonald et al., 2002), bacterial transformation of oil-carbon should be considered as an important process in the fate of GCS-oil slicks with consequences for microbial food web interactions.

### Conflict of interest statement

The authors declare that the research was conducted in the absence of any commercial or financial relationships that could be construed as a potential conflict of interest.
